# Clinical significance of serum and vitreous soluble interleukin-2 receptor in patients with intraocular lymphoma

**DOI:** 10.1186/s12886-022-02677-4

**Published:** 2022-11-10

**Authors:** Kayo Suzuki, Kenichi Namba, Satoru Kase, Yo Ogino, Keitaro Hase, Daiju Iwata, Kazuomi Mizuuchi, Miki Hiraoka, Nobuyoshi Kitaichi, Susumu Ishida

**Affiliations:** 1 Department of Ophthalmology, Faculty of Medicine and Graduate School of Medicine, Hokkaido University, N-15, W-7, Kita-Ku, Sapporo, 060-8638 Japan; 2 Department of Ophthalmology, Health Sciences University of Hokkaido, Sapporo, Hokkaido Japan

**Keywords:** Soluble interleukin-2 receptor, Matrix metalloproteinases, Intraocular lymphoma, Vitreoretinal lymphoma, Central nervous system lymphoma

## Abstract

**Background:**

Intraocular lymphoma (IOL) is a masquerade syndrome that mimics uveitis, making diagnosis difficult. The serum soluble interleukin-2 receptor (sIL-2R), which is cleaved by matrix metalloproteinase (MMP) -2 and MMP-9, has been recognized as a tumor-related biomarker of malignant lymphomas. The aim of this study was to review the reliability of serum and vitreous sIL-2R for distinguishing IOL from uveitis.

**Methods:**

Patients who underwent diagnostic vitrectomy for marked vitreous haze at Hokkaido University Hospital between April 2014 and June 2019 were enrolled. The patients were divided into an IOL group and a uveitis group, according to the pathology of their vitreous samples. The IOL group was further divided at the time of vitrectomy into patients who already had extraocular involvement (IOL with extraocular involvement group) and patients with no evidence of having extraocular involvement (IOL without extraocular involvement group). Serum sIL-2R, and intravitreal sIL-2R, MMP-2, and MMP-9 levels were assessed.

**Results:**

Twenty-five eyes of 25 patients, and 15 eyes of 15 patients were included in the IOL group and uveitis group, respectively. The serum sIL-2R levels were significantly lower in the IOL group than in the uveitis group (*P* < 0.05), and 20.0% and 66.7% in the IOL and the uveitis group showed high sIL-2R value above the normal range. Vitreous sIL-2R tended to be higher in the IOL group than in the uveitis group (*P* = 0.80). Serum sIL-2R was significantly lower in the IOL without extraocular involvement group than in the IOL with extraocular involvement group (*P* < 0.05); 5.9% in the IOL without extraocular involvement group and 50.0% in the IOL with extraocular involvement group showed high sIL-2R value above the normal range. Vitreous sIL-2R, MMP-2, and MMP-9 tended to be higher in the IOL with extraocular involvement group than in the IOL without extraocular involvement group (*P* = 0.30, < 0.05, 0.16).

**Conclusions:**

Serum sIL-2R is often within the normal range in IOL patients. Even if it is within the normal range, the possibility of IOL should be considered. Serum sIL-2R is not a reliable biomarker for IOL, whereas vitreous sIL-2R may be useful for the diagnosis of IOL.

**Supplementary Information:**

The online version contains supplementary material available at 10.1186/s12886-022-02677-4.

## Background

Intraocular lymphoma (IOL) comprises primary IOL and secondary IOL. Primary IOL is further divided into primary vitreoretinal lymphoma and vitreoretinal lymphoma derived from primary central nervous system (CNS) lymphoma. Secondary IOL is the intraocular metastasis of systemic malignant lymphoma [1, 2]. Although IOL is rare, its incidence has gradually increased in recent years, and the prognosis remains poor [3]. Primary vitreoretinal lymphoma metastasizes to CNS at a high rate subsequently. Therefore, correct diagnosis and early treatment before metastasis to the CNS are required to improve the prognosis [3]. However, IOL is a masquerade syndrome that mimics uveitis. There are 2 types of IOL: subretinal lesion type and vitreous haze type, and subretinal lesion type are often accompanied by vitreous haze too [4]. Because IOL frequently shows vitreous haze, it is classified as masquerade syndrome that mimics uveitis. It is difficult to distinguish them from ocular findings especially in the patient showing only vitreous haze, which is not a specific finding for IOL but a common finding in uveitis. Furthermore, responsiveness to corticosteroid treatment is not an important distinguishing factor because even IOL patients sometimes partly respond to corticosteroid therapy. Therefore, reliable biomarkers to distinguish IOL and uveitis are required [5, 6].

The soluble interleukin-2 (IL-2) receptor (sIL-2R) has been found in supernatants of adult T-cell leukemia/lymphoma cell lines [7] and recognized as a tumor-related biomarker of malignant lymphomas [8, 9]. In addition, the serum sIL-2R has recently been reported as a sensitive biomarker for inflammatory diseases, including rheumatoid arthritis, systemic lupus erythematosus, juvenile idiopathic arthritis, and sarcoidosis [10–12]. A high level of sIL-2R has also been reported in the serum of ocular sarcoidosis [13–15]. IL-2R on T cells and B cell lymphoma cells are composed of α, β, and γ chains, and sIL-2R is a soluble form of the α chain of IL-2R, which is cleaved by matrix metalloproteinases (MMP)-2 and MMP-9 [16]. However, the clinical significance of sIL-2R in the diagnosis and metastasis of IOL has not been defined yet. Therefore, the aim of the present study was to investigate the reliability of serum and vitreous levels of sIL-2R in distinguishing IOL from uveitis and estimating the extraocular involvement of primary IOL.

## Methods

### Patients

We retrospectively collected the clinicopathological data including sex, age, ocular findings, and systemic imaging modalities of patients enrolled in this study. Patients were consecutive cases who underwent diagnostic vitrectomy for marked vitreous haze at Hokkaido University Hospital between April 2014 and June 2019. All patients underwent vitrectomy because they were suspected to have IOL. In addition to the marked vitreous haze, they had risk factors of either advanced age, steroid resistancy or subretinal lesions. In patients showing bilateral vitreous haze in which both eyes were operated, the first-operated eye was included in this study. The patients with a final diagnosis of infectious uveitis such as acute retinal necrosis and endophthalmitis were excluded. Written informed consent for use of the collected serum and vitreous in any research was obtained from all the patients at the time of collection. The research protocol of this study including the use of the collected serum and vitreous was explained on the website. This study was conducted in accordance with the guidelines of the Declaration of Helsinki and approved by the institutional review boards of Hokkaido University Hospital (No. 019–0445).

The patients were divided into an IOL group and a uveitis group, according to the results of the pathological examinations. We diagnosed patients with IOL and included them in the IOL group when malignant lymphoid cells were identified pathologically either with vitreous samples obtained by vitrectomy or with biopsy specimens from the extraocular lesions. Cytopathological examination with a vitreous sample was performed using the cell block method, as we previously described [17]. Any presence of extraocular involvement was carefully checked with brain magnetic resonance imaging and positron-emission tomography. The patients with IOL were further divided at the time of vitrectomy into those patients with extraocular involvement, and without extraocular involvement. We included patients in the uveitis group when the patients were ruled out from having IOL with negative results on cytological malignancy, IL-10/IL-6 ratio > 1, and monoclonality of immunoglobulin-heavy chain (IgH) rearrangements. Infectious uveitis was also ruled out from the clinical appearance with the absence of postoperative exacerbation and the negative results of viral PCR examinations. Patients with preoperative trauma, pre-existing macular diseases, vitreous hemorrhage, and diabetes mellitus, which are likely to affect immune mediators in the serum or vitreous humor, were excluded from the study.

### Measurement

The levels of serum sIL-2R obtained at the patients’ first visit were assessed by enzyme-linked immunosorbent assay (LSI Medience, Tokyo, Japan), the normal range of which was < 459 U/ml. We defined the values as positive when they were higher than the normal range.

Undiluted human vitreous samples (500 µl–1000 µl) were collected during 25-gauge pars plana vitrectomy prior to intraocular fluid infusion, as we previously described [17, 18]. The samples were immediately stored at 4ºC, sent to a laboratory within one hour, and stored at -80ºC. The samples were defrosted and centrifuged at 800 G for 10 min, and supernatants were used for the assays. The levels of vitreous sIL-2R, MMP-2, and MMP-9 were measured using a magnetic multiplex bead-based quantitative immunoassay (Magnetic Luminex Assay; R&D systems, Minneapolis, MN, USA).

### Statistical analysis

The differences in the levels of serum and vitreous sIL-2R, MMP-2, and MMP-9 between IOL and uveitis were statistically analyzed using the Mann–Whitney U test. The levels of serum sIL-2R and vitreous sIL-2R, MMP-2 and MMP-9 were statistically analyzed among 3 groups; IOL without extraocular involvement group, IOL with extraocular involvement group, and uveitis group using Kruskal–Wallis test and Dunn test. All data were analyzed using StatPlus software version 7 (AnalystSoft Inc., Alexandria, US). A *P*-value less than 0.05 was considered significant.

## Results

Twenty-five eyes of 25 patients (17 men and 8 women with mean age of 67.9 ± 13.2 years) were included in the IOL groups, and 15 eyes of 15 patients (5 men and 10 women with mean age of 71.9 ± 12.1 years) were included in the uveitis group. All the patients were Japanese adults.

The clinical data of the IOL group are shown in Table 1. All positive tissue biopsies in the IOL groups were suggestive of DLBCL. In addition, 14 of 25 patients had subretinal lesions suggestive of IOL. The IOL group was divided into the IOL without extraocular involvement group (17 eyes) and the IOL with extraocular involvement group (8 eyes).　In the IOL without extraocular involvement group, 7 of 17 patients had received systemic or topical use of corticosteroids such as oral prednisolone, sub-tenon and/or intravitreal injections of triamcinolone acetonide prior to blood tests and vitreous biopsies. All patients in IOL with extraocular involvement group had not received corticosteroids; however, 7 of 8 patients had already undergone chemotherapies for malignant lymphoma until blood tests and vitreous biopsies were performed.

**Table 1:** Clinical data of patients with IOL

Case No	Sex	Age	Laterality	Extraocular involvement	Cell block cytology	IL-10/IL-6 Ratio > 1	IgH
1	F	63	Bilateral	None	+	+	+
2	M	63	Bilateral	None	+	+	+
3	F	80	Bilateral	None	+	+	+
4	M	82	Bilateral	None	+	+	NA
5	M	75	Unilateral	None	+	+	NA
6	F	72	Bilateral	None	+	+	+
7	M	74	Unilateral	None	+	-	+
8	M	82	Bilateral	None	+	+	+
9	M	58	Bilateral	None	+	+	+
10	F	82	Bilateral	None	+	-	+
11	M	58	Bilateral	None	+	+	+
12	F	49	Unilateral	None	+	+	NA
13	M	47	Bilateral	None	+	+	+
14	M	58	Bilateral	None	+	+	+
15	F	92	Bilateral	None	+	+	+
16	M	85	Bilateral	None	+	+	+
17	F	72	Bilateral	None	+	+	+
18	M	82	Unilateral	CNS, Orbit	NA^a^	+	-
19	M	47	Unilateral	CNS, LNs	NA^a^	+	-
20	F	54	Unilateral	CNS	+	+	NA
21	M	64	Bilateral	CNS, LNs	+	+	+
22	M	77	Unilateral	CNS, LNs	+	+	-
23	M	73	Unilateral	CNS	+	+	NA
24	M	63	Bilateral	Testes, Nose, LNs	NA^a^	+	-
25	M	46	Bilateral	LNs	+	-	+

*F* Female, *M* Male, *LNs* Lymph nodes, *CNS* Central nerve system, *IgH* Immunoglobulin-heavy chain rearrangements, *NA* Not available

^a^Malignant lymphoid cells were confirmed with biopsy of extraocular lesions

All of 15 patients in the uveitis group were investigated for etiology at the time of initial presentation with blood, urine, and chest x-rays, including angiotensin-converting enzyme, KL-6, T-spot. TB, syphilis serological tests, anti-HTLV-1 antibody, and beta-D-glucan, resulting in a diagnosis of idiopathic disease in all patients without leading to a specific diagnosis such as sarcoidosis. Furthermore, the group did not include patients showing any of the symptoms that strongly suggest sarcoidosis including elevated angiotensin-converting enzyme, bilateral hilar lymphadenopathy, iris nodules, trabecular meshwork nodules, nodular periphlebitis or optic disc/choroidal granulomas. All patients in the uveitis group including one patient who had a history of malignant lymphoma of inguinal lymph nodes had no subretinal lesions suggestive of IOL. In the uveitis group, 6 of 15 patients had received systemic or topical use of corticosteroids such as oral prednisolone, sub-tenon injections of triamcinolone acetonide prior to blood tests and vitreous biopsies.

**Fig. 1:**
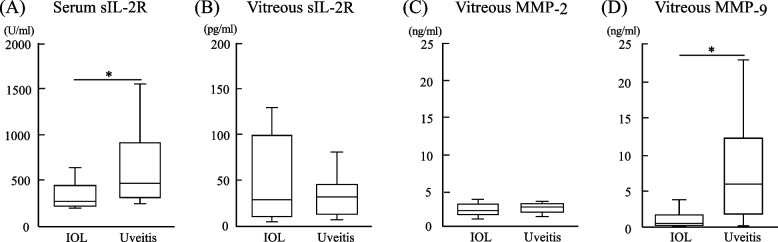
sIL-2R, MMP-2, and MMP-9 in patients with IOL and uveitis. Serum sIL-2R (**A**), and vitreous sIL-2R (**B**), MMP-2 (**C**), and MMP-9 (**D**) in patients with IOL and uveitis. *P < 0.05; Mann–Whitney U test

Figure 1 shows the results of serum sIL-2R and vitreous sIL-2R, MMP-2, and MMP-9 in the IOL and uveitis groups. The serum sIL-2R level was significantly lower in the IOL group (424 ± 482.9 U/ml) than in the uveitis group (659 ± 410.1 U/ml, *P* < 0.05). The level of serum sIL-2R was above the normal range in 20.0% and 66.7% of the IOL and the uveitis groups, respectively. On the other hand, although there was no statistically significant difference, vitreous sIL-2R tended to be higher in the IOL group (180 ± 496.8 pg/ml) than in the uveitis group (35 ± 23.6 pg/ml, *P* = 0.80). Vitreous MMP-2 was not significantly different between the IOL group (2.3 ± 0.79 ng/ml) and the uveitis group (2.5 ± 0.58 ng/ml, *P* = 0.42), whereas vitreous MMP-9 was significantly lower in the IOL group (1.0 ± 1.07 ng/ml) than in the uveitis group (8.3 ± 7.04 ng/ml) (*P* < 0.05).

**Fig. 2:**
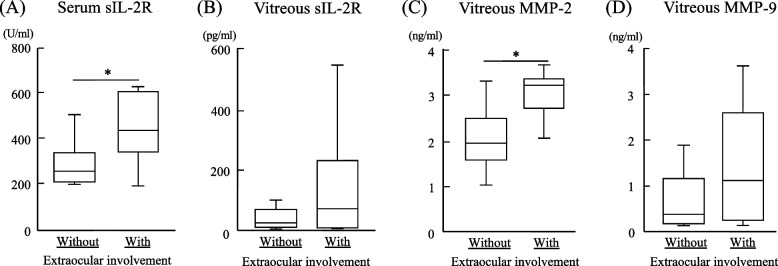
sIL-2R, MMP-2, and MMP-9 in patients with IOL with and without extraocular involvement. Serum sIL-2R (**A**), and vitreous sIL-2R (**B**), MMP-2 (**C**), and MMP-9 (**D**) in patients with IOL with and without extraocular involvement. *P < 0.05; Mann–Whitney U test

Figure 2 shows the levels of serum sIL-2R, vitreous sIL-2R, vitreous MMP-2, and vitreous MMP-9 in the IOL with extraocular involvement group and the IOL without extraocular involvement group. Serum sIL-2R was significantly lower in the IOL without extraocular involvement group (293 ± 97.0 U/ml) than in the IOL with extraocular involvement group (425 ± 56.0 U/ml, *P* < 0.05), and 5.9% in the IOL without extraocular involvement group and 50.0% in the IOL with extraocular involvement group showed high sIL-2R value above the normal range. In addition, although there was no statistically significant difference, vitreous sIL-2R showed a tendency to be higher in the IOL with extraocular involvement group (417 ± 851.1 pg/ml) than in the IOL without extraocular involvement group (69 ± 110.5 pg/ml, *P* = 0.30). Vitreous MMP-2 was significantly higher in the IOL with extraocular involvement group (2.9 ± 0.28 ng/ml) than in the IOL without extraocular involvement group (2.0 ± 0.62 ng/ml, *P* < 0.05), and vitreous MMP-9 also showed a tendency to be higher in the IOL with extraocular involvement group (1.6 ± 1.41 ng/ml) than in the IOL without extraocular involvement group (0.7 ± 0.73, *P* = 0.16), although there was no significant difference.

The results of the comparisons among 3 groups; IOL without extraocular involvement group, IOL with extraocular involvement group, and uveitis group are shown in Supplementary Fig. 1. Serum sIL-2R was significantly higher in the uveitis group than in both IOL with and without extraocular involvement groups (*P* < 0.05). Vitreous sIL-2R was not significantly different among the 3 groups (*P* = 0.60). Vitreous MMP-2 was significantly higher in the IOL with extraocular involvement group than in both the IOL without extraocular involvement group and uveitis groups (*P* < 0.05). Vitreous MMP-9 was significantly higher in the uveitis group than in the IOL without extraocular involvement group (*P* < 0.05).

## Discussion

Serum sIL-2R has been found to be elevated in most types of hematopoietic malignancies, including Hodgkin’s and non-Hodgkin’s lymphomas [13]. As clinical manifestations in malignant lymphoma may mimic a wide variety of diseases, the differential diagnosis of inflammatory disease is crucial in patients presenting with inflammatory symptoms. Several reports have evaluated serum sIL-2R values for the differential diagnosis of lymphoma from other conditions in the clinical setting [13–15].

IOL is a masquerade syndrome that mimics uveitis, making it difficult to diagnose correctly, especially in patients with marked vitreous haze, which is a common ocular finding seen in both IOL and uveitis. However, the clinical usefulness of serum sIL-2R in differentiating between IOL and uveitis remains unclear.　The present study showed that serum sIL-2R was significantly higher in the uveitis group than in the IOL group. Only 20% of patients in the IOL group showed elevated serum sIL-2R, whereas 66.7% of patients in the uveitis group showed an elevation. Although serum sIL-2R is a reliable biomarker for the diagnosis of systemic lymphoma, these results indicates that serum sIL-2R cannot be a biomarker for diagnosis of IOL.

In contrast, although there was no significant difference, vitreous sIL-2R in the IOL group tended to be higher than in the uveitis group. Takeda et al. also reported that vitreous sIL-2R was significantly higher in IOL patients than in uveitis or epiretinal membrane (negative control) patients [19]. These results suggest that sIL-2R may be secreted from the intraocular tissues, and concentrated in the vitreous cavity along with the growth of IOL; however, the amount of sIL-2R leaking out to the serum from the vitreous cavity is too small to increase the level of serum sIL-2R.

The result that the serum sIL-2R level was significantly lower in the IOL group than in the uveitis group needs to be carefully considered. The uveitis patients enrolled in the study were not uveitis patients with common causes, but the idiopathic uveitis patients sufficiently presenting severe vitreous haze to suspect IOL. After IOL was ruled out pathologically, most of the patients might have had ocular sarcoidosis but did not meet the diagnostic criteria of sarcoidosis, even with systemic examinations, and were eventually diagnosed with idiopathic uveitis. We previously reported that the elevation of serum sIL-2R was seen in 25% of IOL patients, 76.4% of ocular sarcoidosis patients, and 5.6% of other uveitis patients [20]. In addition, previous reports have indicated the sensitivity and the specificity of serum sIL-2R levels were 81–98% and 91–99%, respectively, in the diagnosis of ocular sarcoidosis [13–15]. According to the results of these reports, elevated serum sIL-2R is a useful marker for ocular sarcoidosis but not for other uveitis [20–23]. It is unclear why serum sIL-2R was elevated in the uveitis patients in this study. They might have included some ocular sarcoidosis patients who had not been diagnosed yet.

MMP-2 and MMP-9, gelatinases which are capable of degrading type IV collagen, are known to be activated in lymphomas, and their activation is considered to be related to the metastasis of lymphomas [24–26]. We previously showed that the expression of MMP-2 in enucleated eyes and the cell blocks of vitreous samples was significantly higher in secondary IOL than in primary IOL [27]. Similarly, in this study, vitreous MMP-2 was significantly higher and MMP-9 also tended to be high in lymphoma patients with extraocular involvement. These results suggest that the population of lymphoma cells expressing high levels of MMP-2 and MMP-9 among the lymphoma cells with extraocular involvement may have a potentially high metastatic capability to reach the ocular tissue. The elevated levels of vitreous sIL-2R may be the result of the cleavage of IL-2R on the surface of T cells and B cell lymphoma cells with activated MMP-2 and MMP-9.

It has been reported that MMPs enabled T cells to pass through basement membranes by proteolytical cleaving, and that the inhibition of MMP activity was an efficient way to reduce inflammation in uveitis [28, 29]. Vitreous MMP-2 levels were not elevated in either the IOL and uveitis groups in this study, whereas vitreous MMP-9 levels in the uveitis group were significantly higher than in the IOL group. Although the mechanism of sIL-2R elevation in uveitis is still unknown, MMP-2 and MMP-9 may be involved in the migration of lymphocytes in uveitis as well as IOL.

There are certain limitations to the present study. The idiopathic uveitis patients in this study had uveitis of unknown etiology but presenting vitreous haze so thick that IOL was suspected. Therefore, the data on idiopathic uveitis from this study may not be common in generally considered idiopathic uveitis. This may have affected increased serum sIL-2R levels in the uveitis group.

## Conclusions

Serum sIL-2R is often within the normal range in IOL patients. Even if it is within the normal range, it is necessary to consider the possibility of IOL. Meanwhile, vitreous sIL-2R may be useful for diagnosis of IOL. Patients with high sIL-2R, MMP-2, and MMP-9 in the vitreous may have high metastatic capability, and it is necessary to carefully observe these patients for the development of the extraocular involvement.

## Supplementary Information

**Additional file 1: Supplementary Figure 1.** sIL-2R, MMP-2, and MMP-9 in patients with IOL without extraocular involvement, IOL with extraocular involvement, and uveitis. Serum sIL-2R (A), and vitreous sIL-2R (B), MMP-2 (C), and MMP-9 (D) in patients with IOL without extraocular involvement, IOL with extraocular involvement, and uveitis. *P < 0.05; Dunn test.

**Additional file 2: Supplementary Table 1.** sIL-2R, MMP-2, MMP-9 in IOL patients. **Supplementary Table 2.** sIL-2R, MMP-2, MMP-9 in Uveitis patients.

## Data Availability

All data generated or analyzed during this study are included in this published article and its supplementary information files.

## Abbreviations

CNS: Central nervous system
IOL: Intraocular lymphoma
IL-2: Interleukin-2
MMP: Matrix-metalloproteinase
PCNSL: Primary central nervous system lymphoma
sIL-2R: Serum soluble interleukin-2 receptor
