# Supplementary figures and images for: Clinical significance of serum and vitreous soluble interleukin-2 receptor in patients with intraocular lymphoma
Source: BMC Ophthalmol. 2022 Nov 10;22:428. doi: 10.1186/s12886-022-02677-4 (PMC9648008; doi:10.1186/s12886-022-02677-4)

**Supplementary Figure 1.**

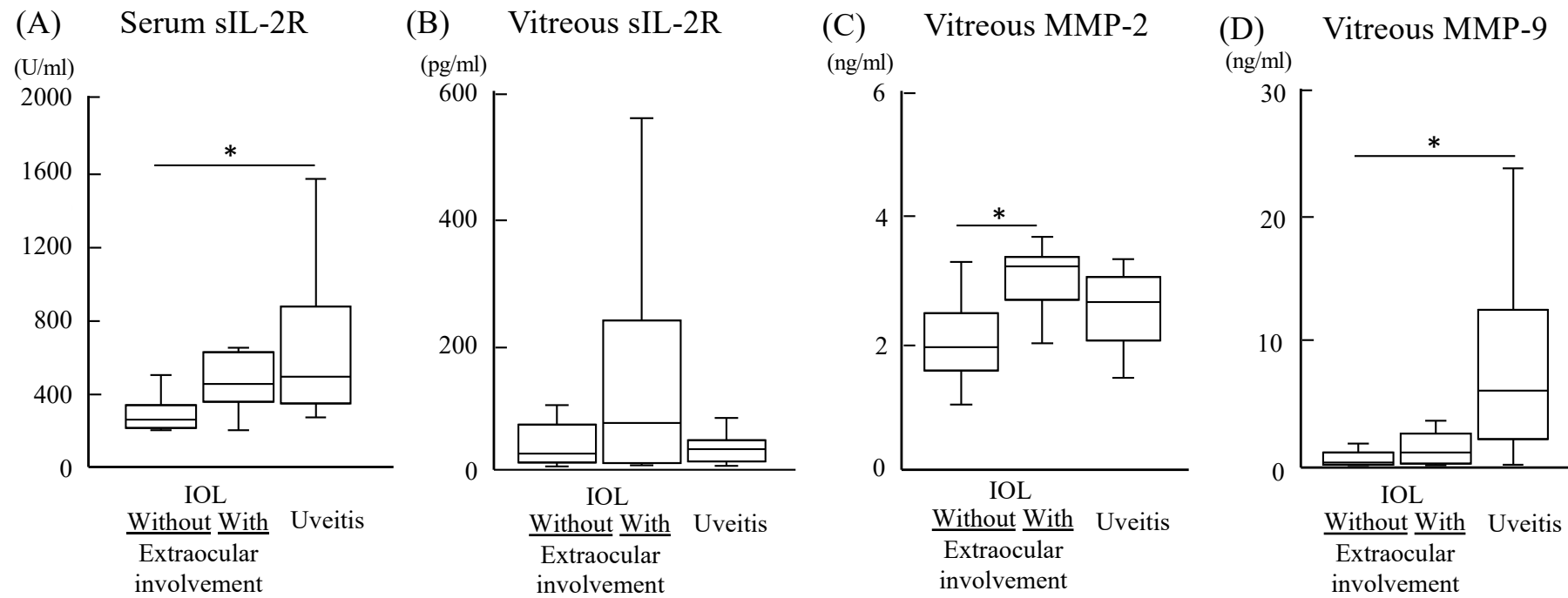

Supplement: Supplementary file 1 — Additional file 1: Supplementary Figure 1. sIL-2R, MMP-2, and MMP-9 in patients with IOL without extraocular involvement, IOL with extraocular involvement, and uveitis. Serum sIL-2R (A), and vitreous sIL-2R (B), MMP-2 (C), and MMP-9 (D) in patients with IOL without extraocular involvement, IOL with extraocular involvement, and uveitis. *P < 0.05; Dunn test. [file 12886_2022_2677_MOESM1_ESM.pdf]
