# Supplementary material for: Clinical significance of serum and vitreous soluble interleukin-2 receptor in patients with intraocular lymphoma
Source: BMC Ophthalmol. 2022 Nov 10;22:428. doi: 10.1186/s12886-022-02677-4 (PMC9648008; doi:10.1186/s12886-022-02677-4)
Supplement: Supplementary file 2 — Additional file 2: Supplementary Table 1. sIL-2R, MMP-2, MMP-9 in IOL patients. Supplementary Table 2. sIL-2R, MMP-2, MMP-9 in Uveitis patients. [file 12886_2022_2677_MOESM2_ESM.docx]

| Case No. | Sex | Age | Laterality |  | Serum |  |  | Vitreous | | |  |
| --- | --- | --- | --- | --- | --- | --- | --- | --- | --- | --- | --- |
|  |  |  |  | Treatments | sIL-2R (U/ml) |  | sIL-2R  (pg/ml) | | MMP-2  (ng/ml) | MMP-9  (ng/ml) | |
| 1 | F | 63 | Bilateral | None | 256 |  | 13.4 | | 1.2 | 0.2 | |
| 2 | M | 63 | Bilateral | None | 334 |  | 13.7 | | 1.3 | 0.2 | |
| 3 | F | 80 | Bilateral | PSL, STTA | 200 |  | 71.1 | | 1.6 | 0.2 | |
| 4 | M | 82 | Bilateral | None | 245 |  | 102.5 | | 1.0 | 0.6 | |
| 5 | M | 75 | Unilateral | None | 441 |  | 14.1 | | 2.7 | 1.2 | |
| 6 | F | 72 | Bilateral | PSL, STTA | 275 |  | 11.1 | | 1.9 | 0.2 | |
| 7 | M | 74 | Unilateral | None | 496 |  | 50.2 | | 2.0 | 1.0 | |
| 8 | M | 82 | Bilateral | IVTA | 306 |  | 270.3 | | 1.9 | 0.6 | |
| 9 | M | 58 | Bilateral | PSL | 208 |  | 9.5 | | 1.4 | 0.2 | |
| 10 | F | 82 | Bilateral | None | 242 |  | 8.0 | | 2.7 | 0.4 | |
| 11 | M | 58 | Bilateral | PSL, STTA | 208 |  | 29.2 | | 2.5 | 0.2 | |
| 12 | F | 49 | Unilateral | None | 200 |  | 30.7 | | 2.5 | 0.2 | |
| 13 | M | 47 | Bilateral | None | 211 |  | 25.8 | | 2.2 | 1.9 | |
| 14 | M | 58 | Bilateral | PSL | 451 |  | 5.2 | | 1.7 | 1.2 | |
| 15 | F | 92 | Bilateral | None | 399 |  | 415.3 | | 3.3 | 0.2 | |
| 16 | M | 85 | Bilateral | None | 272 |  | 6.4 | | 1.7 | 0.4 | |
| 17 | F | 72 | Bilateral | PSL, STTA | 231 |  | 96.7 | | 2.6 | 2.7 | |
| 18 | M | 82 | Unilateral | Chemo | 391 |  | 99.3 | | 3.3 | 0.3 | |
| 19 | M | 47 | Unilateral | Chemo | 613 |  | 2474.0 | | 3.5 | 0.3 | |
| 20 | F | 54 | Unilateral | Chemo | 2655 |  | 129.6 | | 2.9 | 3.2 | |
| 21 | M | 64 | Bilateral | Chemo | 405 |  | 7.1 | | 1.4 | 1.4 | |
| 22 | M | 77 | Unilateral | None | 200 |  | 7.2 | | 3.2 | 0.9 | |
| 23 | M | 73 | Unilateral | Chemo | 642 |  | 48.9 | | 3.3 | 3.7 | |
| 24 | M | 63 | Bilateral | Chemo | 233 |  | 557.1 | | 3.7 | 2.5 | |
| 25 | M | 46 | Bilateral | Chemo | 489 |  | 11.0 | | 2.0 | 0.2 | |

**Supplementary Table 1**. **sIL-2R, MMP-2, MMP-9 in IOL patients**

F, female; M, male. PSL, oral prednisolone; STTA, sub-tenon injection of triamcinolone acetonide; IVTA, intravitreal injections of triamcinolone acetonide; Chemo, Chemotherapies.

**Supplementary Table 2**. **sIL-2R, MMP-2, MMP-9 in Uveitis patients**

| Case No. | Sex | Age | Laterality |  | Serum |  | Vitreous | | |  |
| --- | --- | --- | --- | --- | --- | --- | --- | --- | --- | --- |
|  |  |  |  | Treatments | sIL-2R (U/ml) |  | sIL-2R  (pg/ml) | MMP-2  (ng/ml) | MMP-9  (ng/ml) | |
| 1 | F | 65 | Bilateral | None | 268 |  | 32.6 | 3.3 | 16.1 | |
| 2 | M | 42 | Unilateral | STTA | 1123 |  | 50.5 | 1.8 | 15.6 | |
| 3 | M | 64 | Bilateral | PSL | 333 |  | 11.1 | 1.5 | 11.0 | |
| 4 | F | 65 | Bilateral | None | 268 |  | 31.0 | 3.0 | 12.6 | |
| 5 | F | 90 | Bilateral | None | 1554 |  | 48.5 | 3.1 | 1.9 | |
| 6 | F | 75 | Bilateral | None | 563 |  | 32.5 | 2.3 | 12.4 | |
| 7 | M | 81 | Bilateral | STTA | 809 |  | 82.0 | 2.7 | 2.6 | |
| 8 | M | 66 | Unilateral | PSL | 356 |  | 11.5 | 2.1 | 4.4 | |
| 9 | F | 69 | Bilateral | None | 924 |  | 79.1 | 2.7 | 12.3 | |
| 10 | F | 81 | Unilateral | None | 330 |  | 6.0 | 3.1 | 0.2 | |
| 11 | F | 79 | Bilateral | PSL, STTA | 579 |  | 11.0 | 2.0 | 2.6 | |
| 12 | F | 89 | Bilateral | None | 488 |  | 25.2 | 3.1 | 6.1 | |
| 13 | M | 65 | Bilateral | PSL | 442 |  | 14.0 | 1.7 | 1.9 | |
| 14 | F | 58 | Unilateral | None | 473 |  | 38.0 | 2.7 | 1.1 | |
| 15 | F | 78 | Bilateral | None | 1371 |  | 44.6 | 2.2 | 23.7 | |

F, female; M, male. PSL, oral prednisolone; STTA, sub-tenon injection of triamcinolone acetonide.
